# Physiological and behavioral stress responses to predators are altered by prior predator experience in juvenile qingbo (*Spinibarbus sinensis*)

**DOI:** 10.1242/bio.041012

**Published:** 2019-05-16

**Authors:** Jia-Jia Xu, Shi-Jian Fu, Cheng Fu

**Affiliations:** Laboratory of Evolutionary Physiology and Behavior, Chongqing Key Laboratory of Animal Biology, Chongqing Normal University, Chongqing, 401331, China

**Keywords:** Physiological response, Qingbo (*Spinibarbus sinensis*), Predation experience, Routine metabolic rate, Spontaneous behavior, Boldness

## Abstract

All vertebrates exhibit physiological responses to predator stress and these responses are the basis of appropriate behavioral adaptation. We aimed to identify the physiological and behavioral responses of juvenile qingbo (*Spinibarbus sinensis*) to its natural predator, the southern catfish (*Silurus meridionalis*) and to test whether these responses could be altered by prior predator experience. We measured the routine metabolic rate (RMR), cortisol levels and spontaneous behavior of both predator-naive and predator-experienced qingbo under predator-absent, predator-present and non-predator-present (*Hemibarbus maculatus*) conditions. Predator-naive qingbo showed a typical stress response in the form of increased RMR and cortisol when exposed to predators. Spontaneous activity showed no difference between prior-experience groups or among stimulus conditions when tested alone; however, when tested with a companion, predator-naive qingbo showed increased activity and decreased distance to the stimulus arena under the predator-present condition than they did under the predator-absent condition. Both predator-naive and predator-experienced qingbo showed different physiological and behavioral responses between predatory and non-predatory fish, which suggested that predator-naive qingbo can instinctually discriminate between natural predators and non-predators. Predator-naive qingbo increase their inspection behavior when exposed to a predator compared with the predator-absent condition only when tested with a companion, which is possibly due to decreased predation risk and increased boldness.

## Abstract (precis)

**Summary:** A predator-naive carp can recognize its natural predator, and this recognition can be intensified by prior experience with a predator or the presence of a conspecific.

## INTRODUCTION

Natural selection should favor individuals that alter their physiology and behavior to limit or overcome environmental stressors. The adjustments to stressful conditions are collectively termed the stress response (Bernier and Peter, 2001; Wingfield, 2005; Romero et al., 2009). The hypothalamic-pituitary-interrenal (HPI) axis, through corticosteroid secretion, is an integral mechanism regulating internal homeostasis when vertebrates are faced with a stressor (Wendelaar Bonga, 1997; Lawrence et al., 2017). The main effects of corticosteroid release are associated with the mobilization of energy reserves, which enable the fish to address the increased energy demand associated with stress (reviewed in Gamperl et al., 1994). As the principle corticosteroid in teleosts, in addition to a direct effect, cortisol also has an indirect or permissive effect with other glucoregulatory hormones associated with fish metabolism (reviewed in Gamperl et al., 1994). For instance, fish usually show increased cortisol and an elevated metabolic rate in the presence of actual or model predators (Breves and Specker, 2005; Fürtbauer et al., 2015; Hall and Clark, 2016). However, the intensity or frequency of predation exerts strong selection on the magnitude of the stress response, owing in large part to the detrimental fitness consequences, such as decreased growth, reproduction (reviewed in Wendelaar Bonga, 1997) and immunity (Espelid et al., 1996).

In contrast to the above-mentioned physiological response, spontaneous activity, which is closely related to exploration, inspection and (or) foraging activity, usually decreases in the presence of predators (Barcellos et al., 2007, 2014; Stoks et al., 2003; Balaban-Feld et al., 2018). Because swimming fish leave wakes that contain hydrodynamic and chemical traces in water, decreased spontaneous activity lowers the chances of being detected by predators (Pohlmann et al., 2001) and is one of the most effective anti-predator strategies (Godin, 1997; Biro et al., 2004, 2006). Another reason for decreased activity under predation risk might be that almost all stress responses are energetically costly, whereas a decrease in activity reduces energy expenditure (Lima and Dill, 1990; Brown et al., 2005a). However, decreased activity might entail decreased time spent foraging and engaging in other important activities (Preisser et al., 2005) and thus may potentially affect growth and survival in terms of fish life history (Pangle et al., 2007; Creel and Christianson, 2008). In the presence of predators, a common cyprinid fish species in China, i.e. the qingbo (*Spinibarbus sinensis*), increased rather than decreased its inspection behavior to acquire more information on the predator state (Tang et al., 2018). Thus, the first aim of the present study was to investigate the stress response of the qingbo to its natural predator (southern catfish, *Silurus meridionalis*) (Yang et al., 2011; Gao et al., 2013) as indicated by the cortisol level, routine metabolic rate (RMR) and spontaneous behavior.

Fish individuals have been reported to adjust their physiological and behavioral strategies based on predation risk (Utne et al., 1993; Biro et al., 2003; Conallin et al., 2012; Fu et al., 2015a,b). For example, fish from high-predation populations usually maintain higher cortisol levels and watchfulness than fish from low-predation populations, allowing them to rapidly detect predators. Thus, such individuals have a higher routine energy expenditure than those from low-predation populations (Brown and Braithwaite, 2004; Brown et al., 2005a,b). Furthermore, enhanced respiratory and fast-start escape performance and increased spontaneous movements and boldness have been frequently found in fish populations from high-predation stress habitats relative to those in low-predation habitats (Barton, 2002; Brown et al., 2005b; Bell et al., 2010; Fu et al., 2015a,b). Recent studies found that artificially manipulated short-term predator exposure in the laboratory had profound effects on physiology and behavior, such as elevated energy expenditure, improved swimming capacity and higher survival under predator attack (Lankford et al., 2005; Liu et al., 2016; Fu et al., 2017, 2019). Except for the physiological and behavioral adjustment, the stress response to predators can also be expected to differ between predator-experienced and predator-naive fish. For instance, a previous study found that fish populations exposed to high levels of predation consistently had lower release rates of cortisol in response to a stressor than did conspeciﬁcs sampled at sites with few predators (Archard et al., 2012; Fischer et al., 2014). However, limited data on the influence of short-term predator exposure on the stress response are available for fish species. Thus, the second aim of the present study was to test whether the stress response, as evaluated by routine metabolic rate, cortisol level and measures of spontaneous behavior, was altered by prior short-term predator exposure in the qingbo.

Living in groups often offers increased ability to avoid predators relative that of solitary organisms (Christos, 2017), e.g. when cichlids were given an opportunity to attack free-swimming shoals of guppies, *Poecilia reticulata* (comprised of one to eight individuals), predator hunting success decreased with increasing shoal size (Krause and Godin, 1995). This phenomenon has been observed extensively in social insects, navigating flocks of birds and schools of fish (Christos, 2017); the mechanism underlying this phenomenon might involve the dilution of risk and the ‘many-eyes effect’ (Elgar, 1989; Pitcher and Parrish, 1993; Taraborelli et al., 2012). Thus, predation risk and the associated stress response may differ between solitary and group-living fish. Group living is commonly observed among fish species in nature, and approximately half of all known fish species school for part or all of their lives (Shaw, 1978). However, in most previous studies, the physiological and behavioral responses to predator stress have usually been measured for individual isolated fish, and to date, no comparison of the stress response to predators among different numbers of subject fish has been documented. The third aim of the present study was to identify whether the stress response to predator changes according to the number of qingbo (i.e. with or without a companion).

To fulfill our goals, qingbo, a freshwater cyprinid fish species that prefers group living and is mainly distributed in the upper Yangtze River and its tributaries, was selected as the experimental model. The predation pressure experienced by this fish varies extensively among natural habitats. The southern catfish, a widespread piscivorous fish that shares habitats with qingbo and preys on juvenile qingbo and other cyprinid fishes, was used as a predator in this study (Qin et al., 2016). We measured RMR, cortisol level, measures of spontaneous activity and distance to the stimulus arena in both predator-naive and predator-experienced qingbo under both predator-absent and predator-present conditions and in the presence of a non-predatory fish species (spotted steed, *Hemibarbus maculatus,* a carnivorous fish species that shares the same habitat as qingbo but feeds on zoobenthos).

## RESULTS

### Routine metabolic rate

**Fig. 1.**
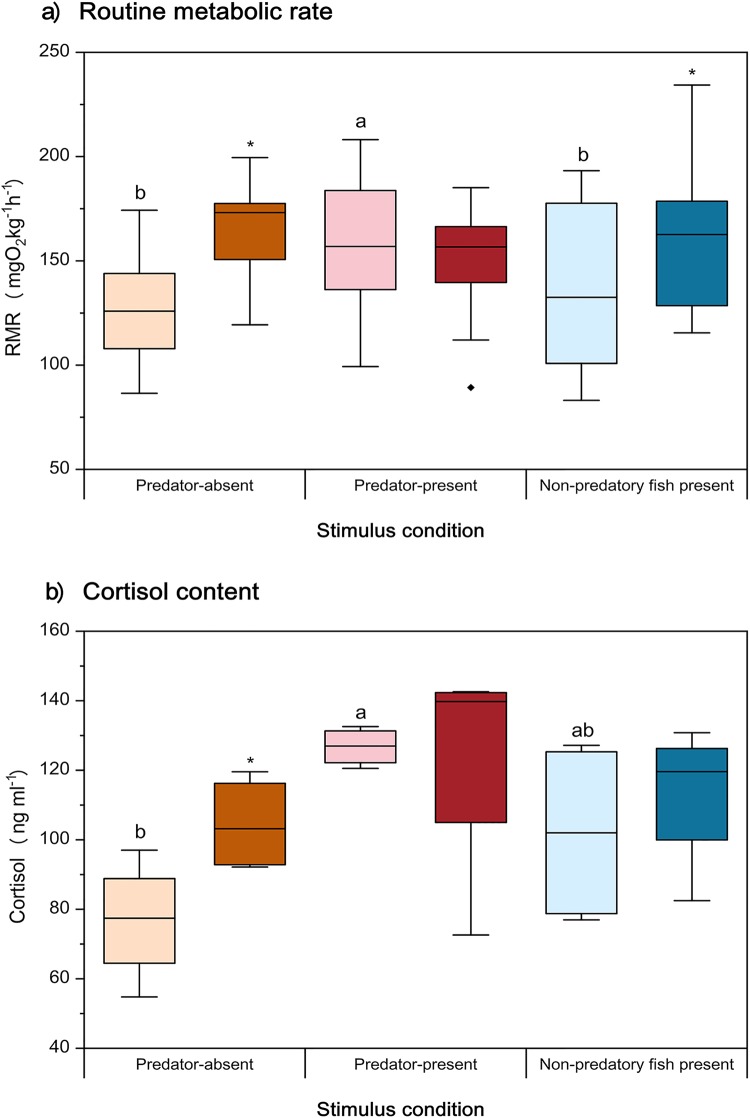
**Effects of prior predator experience and stimulus condition on RMR and cortisol content of qingbo (means±s.e., *n*=20 for RMR and *n*=4 for cortisol content).** Light-colored boxes represent qingbo without a predator experience and dark-colored boxes represent qingbo with a predator experience. Orange, red and blue boxes indicate that the qingbo were measured under the predator-absent, predator-present and non-predatory fish present conditions, respectively. Boxes with different letters indicate significant differences among stimulus conditions within either predator-experienced or predator-naive qingbo. ‘*’ indicates a significant difference between predator-experienced and predator-naive individuals measured under the same stimulus conditions. For the cortisol content (Fig. 1b), see details of the individual data points in Fig. S1.

Prior experience had a significant effect on RMR, and the effect varied significantly among stimulus conditions (interaction: *P*=0.001; Table S1; Fig. 1a). The RMRs of predator-naive qingbo were significantly lower than those of predator-experienced qingbo under both the predator-absent and non-predatory fish (spotted steed)-present conditions (*P*<0.05). However, significant differences were not detected in the predator-present condition. Furthermore, the RMR of predator-naive qingbo was significantly higher under the predator-present condition than under the other two conditions (*P*<0.05).

### Cortisol

Stimulus condition had a significant effect on cortisol content (*P*=0.019; Table S1; Fig. 1b). The cortisol content of predator-naive qingbo measured under predator-present conditions was significantly higher than that measured under predator-absent conditions (*P*<0.05). Furthermore, the cortisol content of predator-experienced qingbo was significantly higher than that of predator-naive individuals when measured under predator-absent conditions (*P*<0.05).

### Spontaneous behavior

**Fig. 2.**
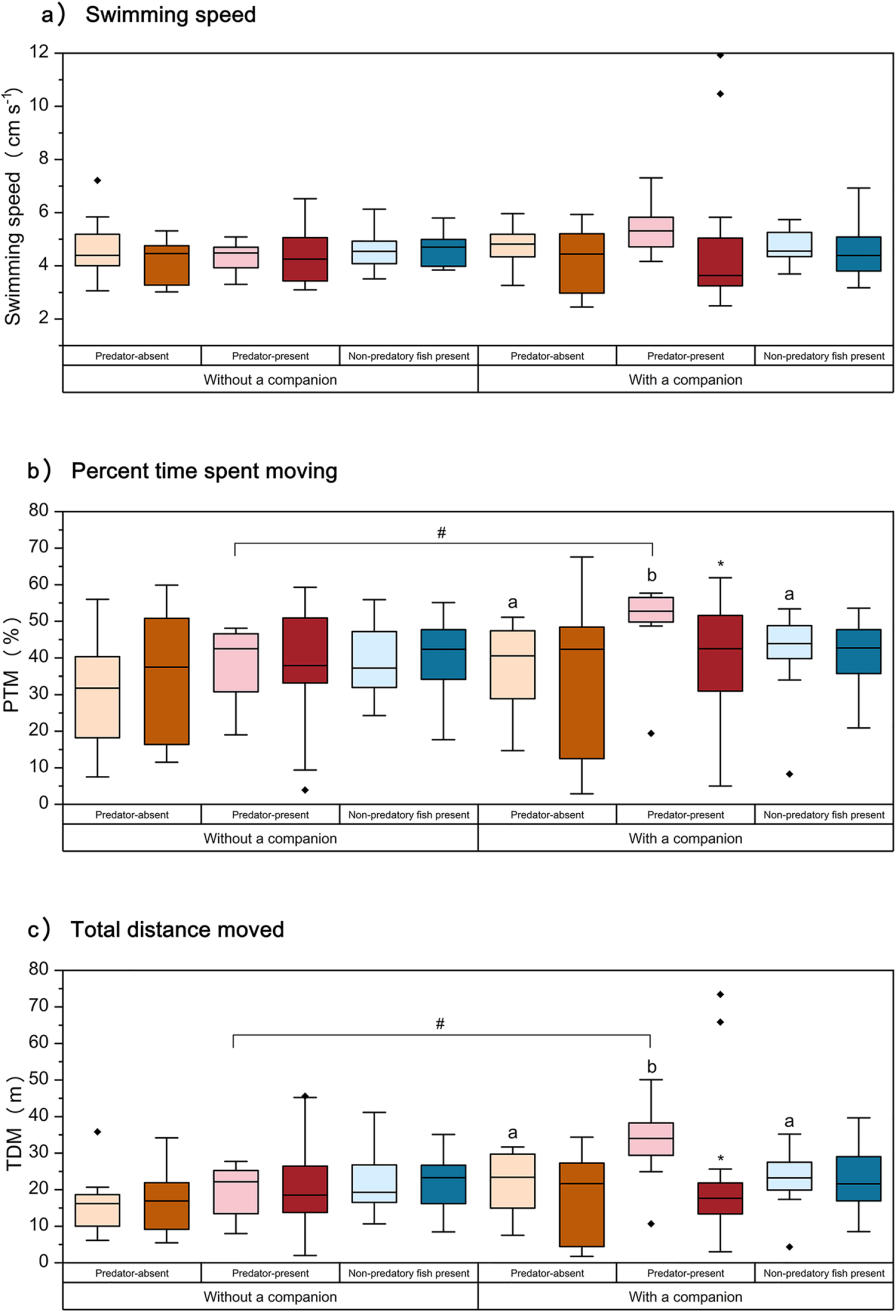
**Effects of prior predator experience, stimulus condition and number of fish on spontaneous activity in qingbo (means±s.e., see** **Table 1** **for information on replicates).** Light-colored boxes represent qingbo without a predator experience and dark-colored boxes represent qingbo with a predator experience. Orange, red and blue boxes indicate that the qingbo were measured under the predator-absent, predator-present and non-predatory fish present conditions, respectively. Boxes with different letters indicate significant differences among stimulus conditions within either predator-experienced or predator-naive qingbo. ‘*’ indicates a significant difference between predator-experienced and predator-naive individuals measured under the same stimulus conditions. ‘#’ indicates a significant difference between predator-naive qingbo measured with or without a companion under predator-present conditions.

Swimming speed was not significantly affected by the prior predator experience treatment, stimulus condition and number of qingbo (Table S2; Fig. 2a). PTM and TDM showed the same trends; i.e. significant differences were not observed in these parameters among the predator experience treatments and the stimulus conditions when individuals were measured individually (Table S2; Fig. 2b,c). However, the PTM and TDM of predation-naive fish with a companion increased significantly under predator-present conditions relative to their values in the other two conditions. This resulted in significantly higher PTM and TDM in these fish than in predator-experienced under the predator-present conditions when measured with a companion (*P*<0.05).

**Table 1.**
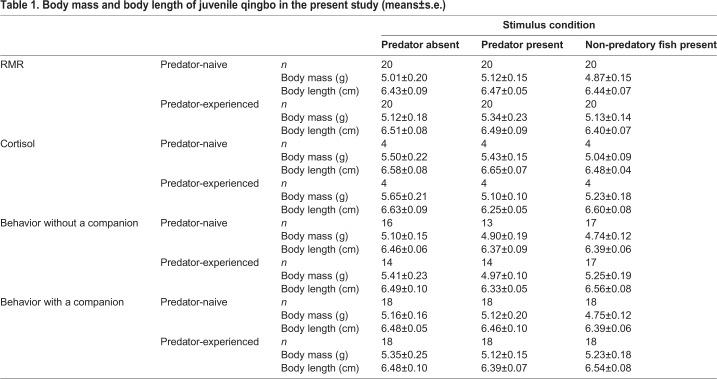
**Body mass and body length of juvenile qingbo in the present study (means±s.e.)**

**Fig. 3.**
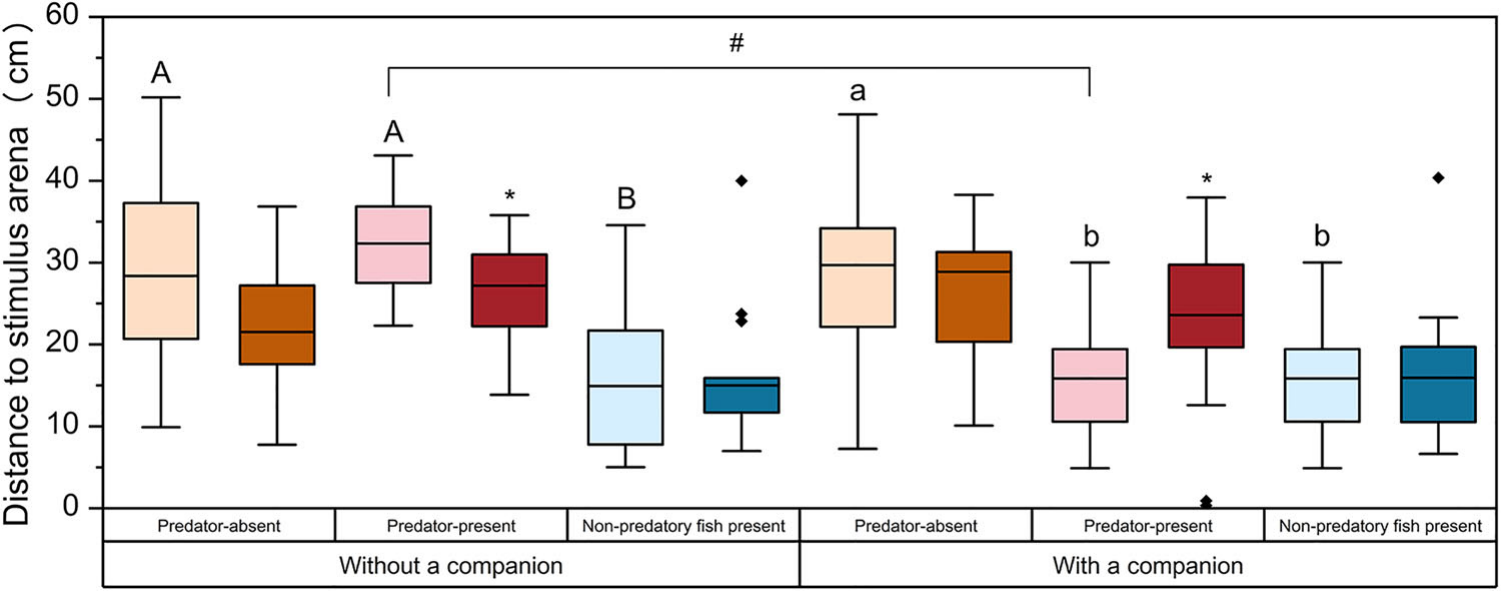
**Effects of prior predator experience, stimulus condition and number of fish on distance to stimulus arena in qingbo (means±s.e., see** **Table 1** **for information on replicates).** Light-colored boxes represent qingbo without a predator experience; and dark-colored boxes represent qingbo with a predator experience. Orange, red and blue boxes indicate that the qingbo were measured under the predator-absent, predator-present and non-predatory fish present conditions, respectively. Boxes with different capitalized letters indicate significant differences among stimulus conditions within predator-naive qingbo measured individually (A,B). Boxes with different lower case letters indicate significant differences among stimulus conditions within predator-naive qingbo measured with a companion (a,b). ‘*’ indicates a significant difference between predator-experienced and predator-naive individuals under the same stimulus conditions. ‘#’ indicates a significant difference between predator-naive qingbo measured with or without a companion under predator-present conditions.

Both stimulus condition and number of qingbo had significant effects on distance to stimulus arena (*P*<0.05); their interaction was also significant (*P*<0.001; Table S2; Fig. 3). When tested individually, predator-naive qingbo under predator-absent and predator-present conditions showed longer distances than those measured under non-predatory fish conditions (*P*<0.05). However, when tested with a companion, predator-naive qingbo under predator-present conditions exhibited significantly lower values of distance to stimulus arena than when tested individually. As a result, among the predator-naive fish, distance to the stimulus arena was not significantly different between the predator-present and non-predatory fish conditions when tested with a companion. Predator experience also showed a significant interaction with number of qingbo (*P*<0.05, Table S2) because predator-naive fish exhibited significantly longer distances than did predator-experienced qingbo when tested individually under predator-present conditions, whereas the opposite pattern was observed when fish were tested with a companion (*P*<0.05).

## DISCUSSION

In nature, fish often need to alter their behavior in response to changing conditions and improve their likelihood of survival. Predation is one of the strongest environmental factors affecting individual survival (Lima, 1998; Lönnstedt et al., 2012; Spiegel et al., 2013; Killen et al., 2016). In the present study, we found that physiological and behavioral adjustments to predator presence were strongly affected by prior predator experience. Predation-naive qingbo typically showed increased cortisol, elevated RMR and increased inspection behavior (only exhibited when measured with a companion) when exposed to predators. However, predator-experienced qingbo showed no differences in physiology and behavior between predator-present and predator-absent conditions, which might be due to either upregulated physiological status in the absence of predators after prior predator exposure or decreased inspection activity, possibly as a consequence of familiarity with the predator. Interestingly, the finding that a behavioral response to predator presence was observed only in individuals tested with a companion suggests that the group size of test fish should be considered in fish behavior research.

### Stress response of predation-naive qingbo following predator exposure

Physiological stress is a general adaptive syndrome of neuroendocrine processes that increase survivorship during life-threatening situations (i.e. stressors) and maintain physiological homeostasis (Wingfield and Ramenofsky, 1999). On the other hand, stress responses directly inhibit growth, development and reproduction and may also impair the digestive efficiency and immunocompetence of animals (reviewed in Hawlena and Schmitz, 2010). The predation-naive qingbo showed a significant 32% increase in RMR and a 36% increase in cortisol in response to predator presence relative to the corresponding predator-absent levels. Elevations of maintenance metabolism and hormone levels in the presence of a predator have been documented in several fish species (Brown et al., 2005a; Millidine et al., 2006; Archard et al., 2012; Breves and Specker, 2005; Fürtbauer et al., 2015; Hall and Clark, 2016), e.g. the juvenile ambon damsel (*Pomacentrus amboinensis*) exhibited significantly improved metabolism when exposed to a visual stimulus of its predator (Hall and Clark, 2016). It has been suggested that elevated hormone levels and RMR maintain the fish in a constant state of predator vigilance (Millidine et al., 2006; Abreu et al., 2018) or as a chronic stress response caused by the predator to the fish, which can occur independent of the vigilance of the fish (Hawlena and Schmitz, 2010). Nonetheless, the increased metabolism should lead to increased energy expenditure while the animal is at rest. In nutrient-limited systems, such increased maintenance costs may compromise important life history attributes, such as growth and reproduction (DuRant et al., 2007; Hawlena and Schmitz, 2010).

In addition to the physiological changes mentioned above, changes in behavior were also observed in predation-naive qingbo, with a 17% increase in PTM, an 8% increase in TDM and a 22% decrease in distance to stimulus arena observed when measured with a companion relative to the corresponding predator-absent levels. This result is inconsistent with many previous studies reporting that spontaneous activity decreases in the presence of predators (Barcellos et al., 2007, 2014; Stoks et al., 2003; Balaban-Feld et al., 2018). This result suggested that the predation-naive qingbo increased rather than decreased their inspection behavior from predator-absent levels in the presence of predators. A similar phenomenon has been found in the poeciliid fish, *Brachyrhaphis episcopi*, in which the populations exposed to high levels of predation were consistently more exploratory and active than conspecifics sampled at sites with few predators (Archard et al., 2012). The increased inspection behavior and improved exploration and activity may increase the opportunities among prey fish to obtain information about the state of the predator and allow prey fish to become more familiar with and avoid predators (Archard et al., 2012).

The number of qingbo showed a significant effect on the behavioral response to predator presence. When predation-naive qingbo were tested individually, there was no significant difference in spontaneous behavior between the predator-absent and predator-present conditions. Thus, the number of qingbo had a strong effect on the stress response to predators. The reason for this finding might involve the higher predation risk and associated lower boldness of solitary individuals than of group-living individuals, as stated in the Introduction (Elgar, 1989; Taraborelli et al., 2012; Foster and Treherne, 1981; Landeau and Terborgh, 1986). This topic should be given more attention in the future, as the results depend on the group size of the subject fish. For fish species that prefer group living, we suggest that two or more fish be accepted as the standard protocol; otherwise, the experimental results might not accurately reflect behavior in the field. Our data show that differences among experiments might arise from variation in environmental conditions (e.g. high versus low predation risk or high versus low food abundance), prior experience (e.g. presence versus absence of prior predator exposure, see details in a later section) and other conditions (e.g. group size of tested fish and size and structure of experimental arena). Furthermore, the stress response to predators might be species-specific as a result of long-term evolution and the specific environmental conditions of species' natural habitats.

Fish usually present high mortality throughout the juvenile phase (Holbrook and Schmitt, 2003). In this phase, juvenile fish must quickly learn to assess the relative danger of situations and respond appropriately to maximize fitness. Such behavior relies on their ability to process information from predator stimuli and discriminate between predatory and non-predatory fish (Hall and Clark, 2016). In the present study, we used spotted steed as a non-predatory control stimulus fish, allowing us to evaluate whether any observed behavioral adjustment in the presence of southern catfish from the predator-absent condition might be due to reasons other than the change in predation risk. The present study revealed that although both southern catfish and spotted steed are of similar size, predation-naive qingbo can distinguish between the two species. This ability was evidenced by the lack of difference in the physiology or behavior of qingbo between the predator-absent and non-predatory-fish conditions. This finding suggested that discrimination of natural predators might be innate in qingbo. Qingbo might identify predators and non-predatory fish based on visual or chemical cues or some combination thereof, while the visual cues may act as a major factor because such visual information is usually of primary importance over short distances (Ward and Mehner, 2010). A lack of a physiological response to a non-predator of similar size to a predator and the ability to rapidly discriminate between similarly sized predatory and non-predatory fishes has been documented previously in other fish species, such as the ambon damsel (*Pomacentrus amboinensis*) (Hall and Clark, 2016). Interestingly, the only difference between the predator-absent and non-predatory-fish conditions was that qingbo showed a shorter distance to the stimulus arena under the latter. This did not represent inspection for predators, as qingbo tested individually also showed a shorter distance to the stimulus arena in the non-predatory-fish condition than in the predator-absent condition. The behavior might reflect seeking protection or increased foraging efficiency from non-conspecific companions (Camacho-Cervantes et al., 2014; Farine et al., 2015). A previous study found that qingbo preferred to associate with larger groups of either conspecifics or heterospecific non-predators in binary choice tests (Xiong et al., 2018).

### Prior predator experience alters the stress response

Compared with the predation-naive qingbo, the predator-experienced qingbo showed no stress response to a predator as evaluated by physiology and behavior measures between the predator-absent and predator-present conditions. The reason for the lack of spontaneous behavior in these fish might be their familiarity with the predator; there may be no need for inspection, and any increased activity would increase predation risk by facilitating detection by the predator. Additionally, the increased activity would also lead to higher energy consumption (Fu et al., 2017). However, the lack of changes in cortisol and RMR was because in the absence of the predator, qingbo with prior exposure to the predator showed elevated cortisol and RMR relative to the levels of predator-naive qingbo. Thus, the variables showed no further change after the introduction of the predator. Increased RMR after short-term (several weeks) acclimation with predators has been frequently found in this species (Liu et al., 2016) and other fish species, such as brown trout (*Salmon trutta*) (Lankford et al., 2005). It has been suggested that increased RMR and cortisol, which lead to elevated alertness, might be related to the reduced response latency of fish with previous predator experience to predators as a result of improved nerve conduction velocity (Killen et al., 2015; Liu et al., 2016; Fu et al., 2017, 2019). In addition, field studies have found that fish from high-predation populations may be under selection to remain in a sustained state of physiological readiness for predator avoidance activity. Such a state would entail high metabolic investment in fueling cardiac and respiratory pumps and maintaining the sensory apparatus (e.g. visual and auditory senses) at elevated acuity (Cooke et al., 2003; Millidine et al., 2006; Fu et al., 2015a,b; Sunadri et al., 2007).

However, both the present study and previous studies (e.g. Millidine et al., 2006) have suggested that the cortisol level combined with the RMR of fish may not increase in some inessential situation, e.g. a fish that shares a habitat with predators, although its RMR may not increase if a shelter is found nearby (Millidine et al., 2006) because a trade-off may exist between predator avoidance and energy balance (e.g. the energy for growth, locomotion and reproductive investment) (Guderley and Pörtner, 2010; Killen et al., 2015). Furthermore, most antipredator strategies present an energy cost (Biro et al., 2006); e.g. hiding in the shelter may lead to a loss of opportunity for obtaining food (Fu et al., 2015a). Thus, the stress response combined with metabolism may represent an important moderator of fish behavioral responses to their predators.

Thus, regarding the anti-predator strategy adopted by qingbo in the present study, although increases in cortisol during prey–predator interactions are potentially effective for mitigating predator stress, chronically elevated levels may have adverse effects, such as decreased immunity (Espelid et al., 1996) and growth (Davis, 2010), and lead to an overall greater mortality rate (Davis, 2010). Recent studies on cortisol reported that the experimental cortisol elevation (cocoa butter containing cortisol implanted into the peritoneal cavity) did not alter predator avoidance behaviors (shelter use and activity) or the predation rate of several fish species (Lawrence et al., 2018, 2019), which suggested that the cortisol elevation under experimental conditions may be different from that under situations in which stress responses are caused by a real predator. The underlying mechanism requires additional investigation.

In conclusion, the predation-naive qingbo showed a typical stress response in the form of increased cortisol and upregulated metabolism, possibly due to upregulated alertness. The fish also showed enhanced inspection behavior as indicated by increased activity and a shorter distance to the stimulus arena when tested with a companion. However, qingbo with prior predator experience showed no behavioral and endocrinal responses to the presence of predators, possible due to their familiarity with the predator and the upregulated alertness already present as a consequence of the prior exposure treatment. The chronic stress response might result in decreased immunity or reduced responses to additional acute stressors. The present study clearly demonstrated that prior predation had a strong effect on the stress response to predators. More importantly, the results showed that group size is a crucial factor to consider in the study of fish behavior, at least for group-living fish, such as the qingbo.

## MATERIALS AND METHODS

### Experimental animals and acclimation

Experimental juveniles of spotted steed (150–200 g, *n*=20) and southern catfish (150–200 g, *n*=20) were obtained from a local aquatic product market (Shapingba District, Chongqing, China). Experimental juvenile qingbo (Table 1) were obtained from a local fisheries hatchery (Hechuan District, Chongqing, China). The juvenile qingbo had never been exposed to spotted steed or southern catfish before the experiment. All the three fish species were maintained in fully aerated tanks of dechlorinated water (approximately 250 l and 150–200 fish per tank) for 1 month of acclimation before the experiment began. The qingbo and spotted steed were fed to satiation with a commercial diet once daily at 10:00 h. The southern catfish were fed to satiation with cutlets of freshly killed silver carp (*Hypophthalmichthys molitrix*) once daily at 10:00 h. Uneaten food and feces were removed with a siphon 1 h after feeding. All tanks had approximately 10% of the total water volume replaced daily. The water temperature was maintained at 20±1°C and the oxygen content of the water was maintained above 7.0 mg l^−1^. The photoperiod was 12 light:12 dark. This study was approved by the Animal Care and Use Committee of the Key Laboratory of Animal Biology of Chongqing (permit number: Zhao-20170912-01) and performed in strict accordance with the recommendations in the Guide for the Care and Use of Animal at the Key Laboratory of Animal Biology of Chongqing, China.

### Experimental design

**Fig. 4.**
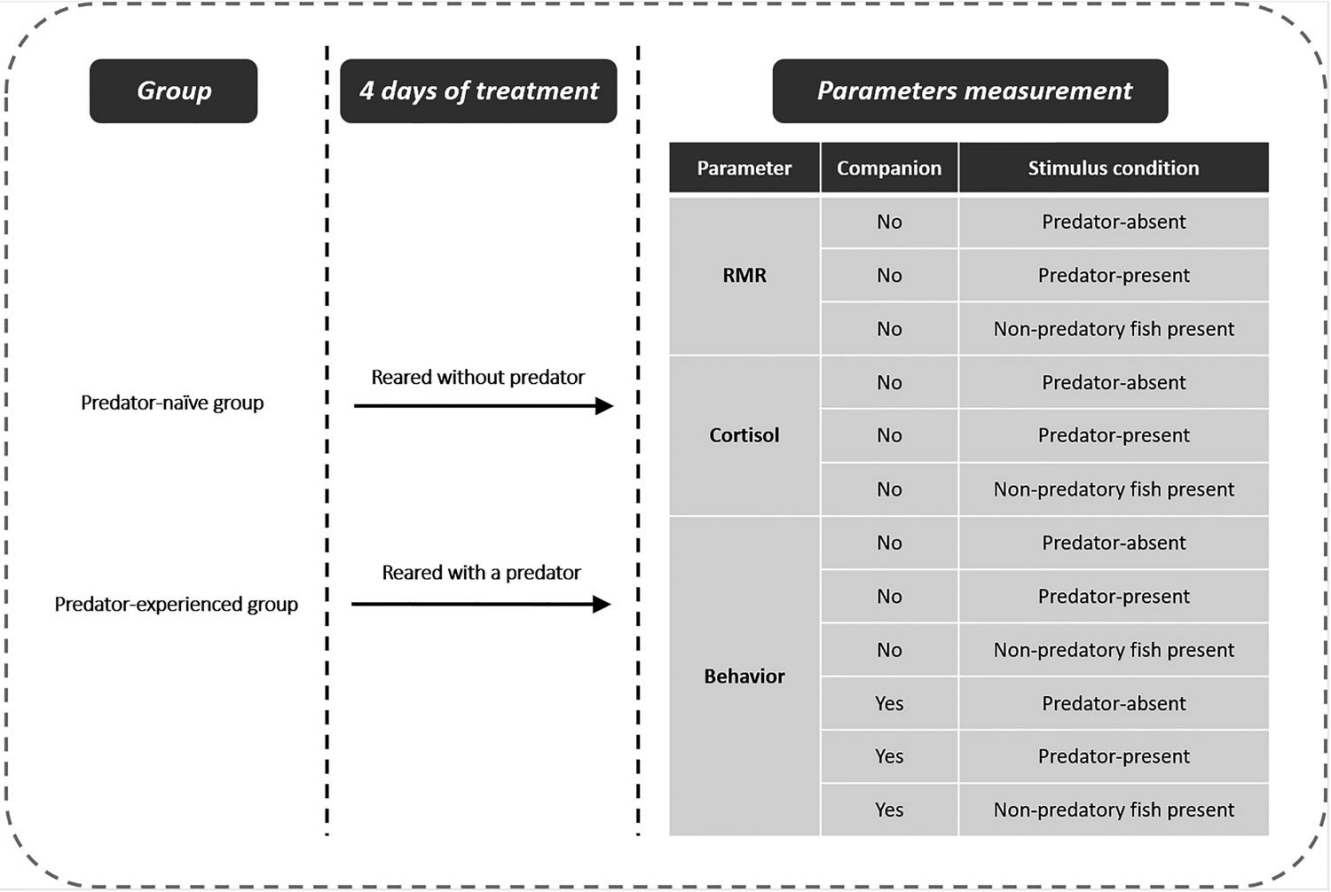
**Scheme of the experimental** **design of the study.** Under ‘Parameter measurements’, companion ‘No’ (or ‘Yes’) means without (or with) a companion present when the parameters of the individual fish were measured.

Qingbo were randomly divided into predator-naive (non-predator-exposure, *N*=200) and predator-experienced (predator-exposure, *N*=200) groups (see details in Fig. 4). The qingbo in both groups were treated for 4 days. During treatment, the qingbo in the predator-experienced group were reared together with the predator, i.e. the southern catfish. A transparent net was used to separate the predator and qingbo. Additionally, two to three non-experimental qingbo were added to the predator side of the tank as prey, allowing the test qingbo obtain predation experience. In the predator-naive group, a transparent net was similarly used to divide the tank, but nothing was added to the side opposite the qingbo. The other rearing conditions were the same as those in the acclimation period. After 4 days of treatment, three groups (Table 1, Fig. 4) of 20 individuals from each treatment were selected for the measurement of RMR in a sealed chamber under predator-absent, predator-present (visual contact) or non-predatory fish conditions (see below for details). In addition, 12 individuals (Table 1) from each of the predator-experienced and predator-naive groups were selected for the measurement of cortisol under predator-absent, predator-present or non-predatory fish present conditions (see details below). Then, an additional six groups (Table 1) of 18 individuals were selected from each treatment, and measures of spontaneous activity and distance to the stimulus arena were measured individually (or with a companion) under predator-absent, predator-present or non-predatory fish conditions (see below for details of measurements). None of the fish were reused for different measurements in this study.

### Experimental procedure

#### Metabolism

The oxygen consumption of both predator-naive and predator-experienced fish was measured by a flow-through respirometer (see structure in Fu et al., 2005). The respirometer chambers were submerged into the water of the tank, and the area outside of the respirometer chambers either remained empty of stimulus fish (predator-absent condition) or contained a southern catfish (predator-present condition) or spotted steed (of similar size as the southern catfish; non-predatory fish condition). Before the measurements were collected, the experimental fish were transferred into a transparent tubular respirometer chamber (350 ml) after 24 h of fasting and allowed to acclimate for another 36 h. During transfer, an individual fish was gently netted from their holding tank into a plastic beaker without exposure to air and then released carefully into the respirometer chamber. The oxygen consumption rate was measured eight times at 90-min intervals starting at 08:00 h. The average value of eight measurements was taken as the RMR of the qingbo. The following formula was used to calculate ṀO_2_ (mg O_2_ kg^−1^ h^−1^):
(1)
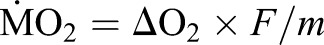
where ΔO_2_ is the difference in the oxygen concentration (mg O_2_ L^−1^) between the experimental chamber and the control chamber (the chamber without fish); *F* is the water flow rate in the experimental chamber (l h^−1^); and *m* is the body mass of the fish (kg). The dissolved oxygen concentration was measured at the outlet of the chamber using an oximeter (HQ30d, Hach Company, Loveland, CO, USA). The flow rate of water through the respirometer chamber was measured by collecting the water that was expelled from each chamber. The measurement conditions (e.g. water temperature and dissolved oxygen level) were the same as those in the acclimation condition.

#### Cortisol

**Fig. 5.**
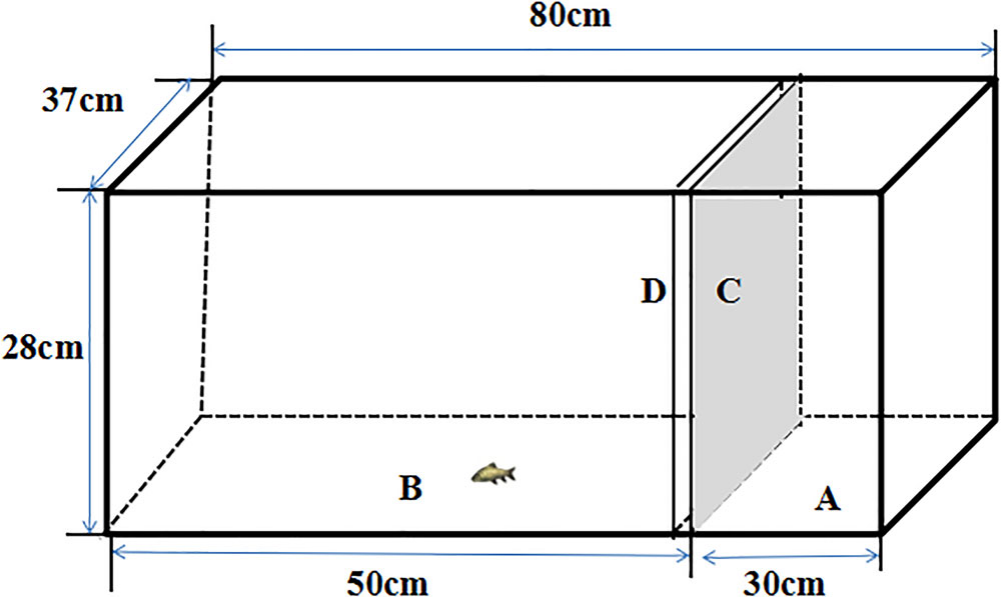
**Experimental setup for measurements of cortisol and spontaneous behavior in the present study.** A, stimulus arena; B, experimental arena; C, opaque sheet; D, transparent sheet.

A rectangular aquarium (80×37×28 cm, Fig. 5) was used to measure cortisol. The aquarium was divided into two sections (an experimental arena and a stimulus arena) by a removable transparent sheet. Fish (Table 1) were individually transferred into a beaker (1 l, water depth 8 cm) that was then placed in the experimental arena of the aquarium, and the fish was allowed to acclimate and exposed to different stimulus conditions for 36 h. The beaker was transparent so that the fish in one beaker could see the fish in the other beakers as well as the condition of the stimulus arena. During this time, the beaker was aerated and the stimulus arena was left empty or a southern catfish or spotted steed was introduced. After 36 h of acclimation and exposure, tricaine methanesulfonate (MS-222 concentrated solution) was noiselessly added to the beaker with a tube to euthanize the fish (concentration: 0.1 g l^−1^) without disturbance. Such an operation was performed to maintain a similar rhythm as the RMR measurement and to avoid the stress response when catching the fish for euthanasia. The tail was immediately removed via scalpel, and blood from the caudal artery and vein was sampled using a capillary tube. The blood samples were placed in centrifuge tubes lined with anticoagulant (heparin sodium salt) and then centrifuged at 3000 r min^−1^ for 10 min. The supernatants were stored at −80°C for further analysis. The cortisol contents were measured using a radio immunoenzyme-linked assay with a cortisol ELISA (enzyme linked immunosorbent sorbent assay) kit purchased from Cayman (USA), and the concentration was measured in units of ng ml^−1^. The assay we used in this study is based on the competition between cortisol and cortisol-acetylcholinesterase (AChE) conjugate (cortisol tracer) for a limited number of cortisol-specific mouse monoclonal antibody binding sites. The assay has a range of 6.6–4000 pg ml^−1^ and a sensitivity (80% B/B_0_) of approximately 35 pg ml^−1^. Four individuals were measured under each measurement condition. The inter- and intra-assay coefficients of variation were 4.2–9.4% and 5.6–6.7%, respectively.

#### Spontaneous behavior

Spontaneous behavior of fish (*n*=18) was measured in the rectangular aquarium (Fig. 5). The qingbo were placed in the experimental arena and either the stimulus arena was left empty, or a southern catfish or spotted steed was introduced into the area. Both the stimulus fish (southern catfish or spotted steed) and the qingbo were allowed to acclimate for 10 min. During this acclimation time, the experimental arena and stimulus arena were divided by two sheets (one opaque and the other transparent). Then, the opaque sheet was removed and the qingbo were recorded by a video camera (Logitech C310, 15 frames s^−1^) for 20 min (Fu et al., 2017), which was installed directly above the aquarium. The fish trajectory in each frame was digitized using the software idTracker (Pérez-Escudero et al., 2014), which automatically tracked the position of each fish in each trial and provided all x and y coordinates of each fish in each video (Miller and Gerlai, 2012; Killen et al., 2016). For the fish with a companion, one of the two fish per pair was selected randomly as the focal fish for further analysis. The raw trajectories were smoothed using a weighted average method with a window width of 0.5 s; this was performed because the trajectories were noisy due changes in body shape during recording and errors from the tracking device (Miller and Gerlai, 2012). Of the 216 videos, 17 failed to be digitized by idTracker and were excluded from analysis (see details in Table 1). The spontaneous behavior variables were as follows: (1) swimming speed (cm s^−1^): a fish was considered moving when its swimming speed was higher than 1.75 cm s^−1^, otherwise it was considered resting; (2) percentage of time spent moving (PTM, %): the percentage of time the individual's speed was higher than 1.75 cm s^−1^; and (3) total distance moved (TDM, cm): the total distance covered by the individual over the whole measurement period (20 min).

In addition, we recorded distance to stimulus arena (cm), calculated as the average distance between the qingbo individual and the transparent sheet.

### Data handing and analysis

SPSS Statistics 17.0 (SPSS, Chicago, IL, USA) was used for statistical analysis. The effects of prior predator experience and stimulus condition on the RMR and cortical content of juvenile qingbo were analyzed using general linear models incorporating two-way analysis of variance (ANOVA). The effects of predator experience, stimulus condition and number of qingbo on spontaneous activities (i.e. spontaneous swimming speed, PTM and TDM) and distance to stimulus arena were tested by a general linear model incorporating three-way multivariate analysis of variance (MANOVA). Differences in variables between the predator-experienced and predator-naive individuals (or between the individuals with and without a companion) were evaluated by independent-samples *t*-tests, whereas differences in variables among the three stimulus conditions within each experience group and within each grouping type (with or without a companion) were compared using Duncan's multiple comparisons test. All data are presented as the means±s.e., and *P*<0.05 was used as the level of statistical significance.

## Supplementary Material

Supplementary information
