# Supplementary material for: Physiological and behavioral stress responses to predators are altered by prior predator experience in juvenile qingbo (Spinibarbus sinensis)
Source: Biol Open. 2019 May 16;8(5):bio041012. doi: 10.1242/bio.041012 (PMC6550089; doi:10.1242/bio.041012)
Supplement: Supplementary information [file biolopen-8-041012-s1.pdf]

Fig. S1 Cortisol content

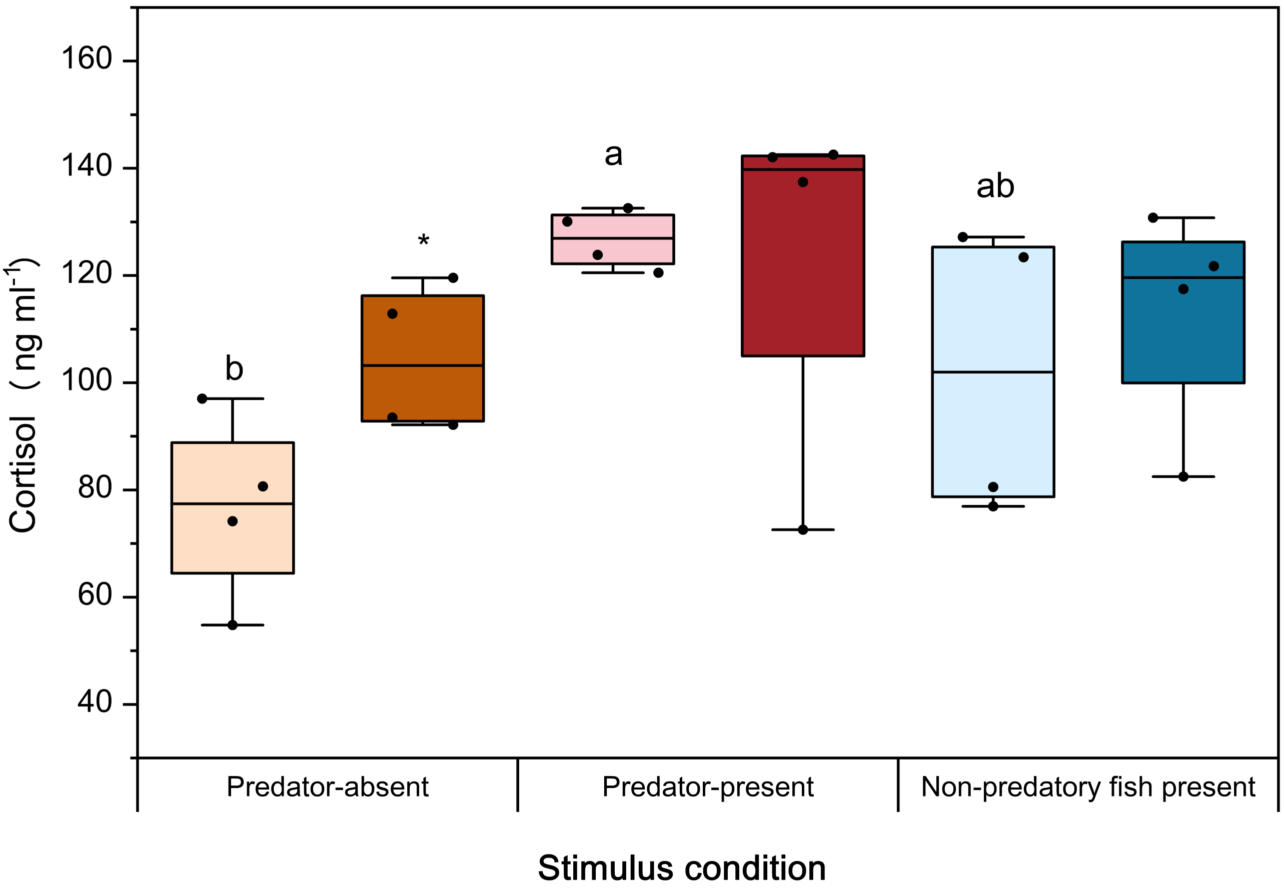

Fig. S1 Effects of prior predator experience and stimulus condition on cortisol content of qingbo (means ± S.E., n = 4). Light-colored boxes represent qingbo without a predator experience; and dark-colored boxes represent qingbo with a predator experience. Yellow, red and blue boxes indicate that the qingbo was measured under the predator-absent, predator-present and non-predatory fish present conditions, respectively. a, b: Boxes with different letters indicate significant differences among stimulus conditions within either predator-experienced or predator-naive qingbo; and * indicates a significant difference between predator-experienced and predator-naive individuals measured under the same stimulus conditions. The individual data points were provided in the plot.

Biology Open • Supplementary information

Table S1 Effects of prior predator experience and stimulus condition on routine metabolic rate and cortisol content in qingbo as determined from two-way analysis of variance (ANOVA).

	Experience effect	Stimulus effect	Interaction effect
Routine metabolic rate (RMR)	$F_{1,119}$ = 11.579	$F_{2,119}$= 0.926	$F_{2,119}$ = 7.052
	P = 0.001*	*P* = 0.399	*P* = 0.001*
Cortisol content	$F_{1,23}$ = 1.797	$F_{2,23}$= 5.017	$F_{2,23}$ = 1.005
	P = 0.197	*P* = 0.019*	*P* = 0.386

* significant (*P* < 0.05)

Table S2 Effects of prior predator experience, stimulus condition and number of qingbo on spontaneous activity and distance to stimulus arena in qingbo based on three-way multivariate analysis of variance (MANOVA).

	Experience (E) effect	Stimulus (S) effect	Fish number (N) effect	E × S effect	E × N effect	S × N effect	E × S × N effect
Swimming speed	$F_{1,187}$=3.197 *P*=0.075	$F_{2,187}$=0.766 *P*=0.466	$F_{1,187}$=3.040 *P*=0.083	$F_{2,187}$= 0.784 *P*=0.458	$F_{1,187}$=1.532 *P*=0.217	$F_{2,187}$=0.759 *P*=0.175	$F_{2,187}$=0.408 *P*=0.665
Percent time spent moving (PTM)	$F_{1,187}$=0.413 *P*=0.521	$F_{2,187}$=6.407 *P*=0.002*	$F_{1,187}$=4.754 *P*=0.030*	$F_{2,187}$=1.445 *P*=0.238	$F_{1,187}$=3.940 *P*=0.049*	$F_{2,187}$=0.612 *P*=0.543	$F_{2,187}$=0.645 *P*=0.526
Total distance moved (TDM)	$F_{1,187}$=1.827 *P*=0.178	$F_{2,187}$=6.464 *P*=0.002*	$F_{1,187}$=7.943 *P*=0.005*	$F_{2,187}$=0.989 *P*=0.374	$F_{1,187}$=5.170 *P*=0.024*	$F_{2,187}$=1.555 *P*=0.214	$F_{2,187}$=1.204 *P*=0.302
Distance to stimulus arena	$F_{1,187}$=1.112 *P*=0.293	$F_{2,187}$=28.336 *P*<0.001*	$F_{1,187}$=5.034 *P*=0.026*	$F_{2,187}$=2.062 *P*=0.130	$F_{1,187}$=5.703 *P*=0.018*	$F_{2,187}$=8.918 *P*<0.001*	$F_{2,187}$=2.511 *P*=0.084

* significant (*P* < 0.05)
